# Memory Problems During COVID in Low-Income Older Adults

**DOI:** 10.1093/geroni/igab046.751

**Published:** 2021-12-17

**Authors:** Faika Zanjani

**Affiliations:** Virginia Commonwealth University, Richmond, Virginia, United States

## Abstract

Prevention, with widespread lifestyle risk reduction at the community-level, is considered an effective method to decrease Alzheimer’s disease (AD). Diverse low-income older adults in Virginia managing either diabetes/cardiovascular symptoms, were offered weekly lifestyle telephone-health coaching for 12-weeks, providing education, motivations, self-efficacy, and referral services for AD lifestyle risk. Participants provided positive anecdotal feedback and the need for continued health coaching during COVID-19. Thirty participants (predominantly African American/Black female) consented for continued health coaching during the pandemic with 47% reporting memory problems. Findings indicated poorer health status associated with reporting memory problems for poor physical health days, poor mental health days, total mental/physical health poor days, sad days, worried days, tired days, feelings of emptiness, feelings of rejection, feelings of failure, little interest/pleasure, and feeling down. This preliminary work creates the impetus for future large-scale AD prevention investigations to improve the lives of AD-risk, low-income, diverse older adults reporting memory problems.

